# Oxido- and Dioxido-Vanadium(V) Complexes Supported on Carbon Materials: Reusable Catalysts for the Oxidation of Cyclohexane

**DOI:** 10.3390/nano11061456

**Published:** 2021-05-31

**Authors:** Manas Sutradhar, Marta A. Andrade, Sónia A. C. Carabineiro, Luísa M. D. R. S. Martins, Maria de Fátima C. Guedes da Silva, Armando J. L. Pombeiro

**Affiliations:** 1 Centro de Química Estrutural, Instituto Superior Técnico, Universidade de Lisboa, Av. Rovisco Pais, 1049-001 Lisboa, Portugal; marta.andrade@tecnico.ulisboa.pt (M.A.A.); fatima.guedes@tecnico.ulisboa.pt (M.d.F.C.G.d.S.); pombeiro@tecnico.ulisboa.pt (A.J.L.P.); 2 Laboratory of Catalysis and Materials (LCM), Associate Laboratory LSRE-LCM, Faculty of Engineering, University of Porto, 4200-465 Porto, Portugal; 3 LAQV-REQUIMTE, Departamento de Química, Faculdade de Ciências e Tecnologia, Universidade NOVA de Lisboa, Largo da Torre, 2829-516 Caparica, Portugal; 4 Research Institute of Chemistry, Peoples’ Friendship University of Russia (RUDN University), 6 Miklukho-Maklaya Street, 117198 Moscow, Russia

**Keywords:** oxidovanadium(V) complexes, aroylhydrazone, X-ray structure, microwave-assisted oxidation, carbon materials, heterogeneous catalysis

## Abstract

Oxidovanadium(V) and dioxidovanadium(V) compounds, [VO(OEt)L] (**1**) and [Et_3_NH][VO_2_L] (**2**), were synthesized using an aroylhydrazone Schiff base (5-bromo-2-hydroxybenzylidene)-2-hydroxybenzohydrazide (H_2_L). They were characterized by elemental analysis, Fourier-transform infrared spectroscopy (FT-IR), (^1^H and ^51^V) nuclear magnetic resonance (NMR), electrospray ionization mass spectrometry (ESI-MS) and single crystal X-ray diffraction analyses. Both complexes were immobilized on functionalized carbon nanotubes and activated carbon. The catalytic performances of **1** and **2**, homogenous and anchored on the supports, were evaluated for the first time towards the MW-assisted peroxidative oxidation (with *tert*-butylhydroperoxide, TBHP) of cyclohexane under heterogeneous conditions. The immobilization of **1** and **2** on functionalized carbon materials improved the efficiency of catalytic oxidation and allowed the catalyst recyclability with a well-preserved catalytic activity.

## 1. Introduction

Vanadium compounds are active catalysts towards several oxidative organic transformations [1,2,3,4,5,6,7,8,9,10,11,12]. They are often used as homogeneous catalysts and can exhibit high activity (in terms of yield) and enantioselectivity [1,2,3,4,5,6,7,8,9,10,11,12]. However, the homogenous catalytic systems present some limitations concerning catalyst separation from the reaction products, recycling and potential application to continuous flow processes. These shortcomings can be overcome by anchoring the complexes onto solid supports, and that combination can provide both the useful properties of the homogeneous catalysts and the advantages of the heterogeneous systems [13,14,15,16,17]. The well-known positive effects of immobilization of complexes on solid supports include the confinement effect, site isolation, prevention of dimerization and cooperative effect of support, among others [18]. The use of carbon materials as supports has additional advantages, given their tunable texture and surface chemistry, able to fit the envisaged applications [17,18,19,20,21,22,23]. Several carbon supports, such as activated carbon, carbon nanotubes, mesoporous carbon xerogels, graphenes, nanodiamonds, etc., have been used for this purpose [13,14,15,16,17,18,19,20,21,22,23]. Supported oxidovanadium complexes were reported as being catalytically active towards selective oxidation reactions, such as alkanes and alcohols oxidation, styrene epoxidation, oxo-bromination, sulfur oxidation and others [1,2,8,13,14]. The most widely used supports in this context are silicas and zeolites, while examples of oxidovanadium complexes anchored on carbon supports are still scarce in the literature [4,24,25,26,27,28,29,30,31,32,33,34].

In a previous work, we reported the immobilization of six oxidovanadium(V) complexes on various carbon supports (carbon xerogels, activated carbons and carbon nanotubes) and studied their catalytic activities towards oxidation of alkanes and alcohols [13], and recognized advantages of the use of adequately treated carbon nanotubes as supports. In pursuit of our interest in designing metal complexes derived from aroylhydrazones as catalysts for oxidation reactions [4,13,35,36,37,38,39,40,41,42,43,44], herein, we report the synthesis and characterization of an oxidovanadium(V) and a dioxidovanadium(V) complex derived from the aroylhydrazone Schiff base (5-bromo-2-hydroxybenzylidene)-2-hydroxybenzohydrazide (H_2_L) [36]. Moreover, aiming at obtaining an improved catalytic activity for the peroxidative oxidation of cyclohexane, we decided also to explore the activity of these two V(V) compounds heterogenized on different carbon materials (which thus would be compared) as catalysts for the microwave-assisted oxidation of cyclohexane with aqueous *tert*-butylhydroperoxide (TBHP).

Both compounds were anchored on activated carbon (AC) and multiwalled carbon nanotubes (CNT) with different surface chemistries, as received (AC and CNT), treated with nitric acid (-ox) or treated with nitric acid and subsequently with NaOH (-ox-Na). The use of MW irradiation as an alternative energy source is quite appealing for organic synthesis, due to superior efficiency and selectivity, as well as for its environment-friendly nature. In light of previous studies, enhanced product yields are anticipated for an MW-assisted catalytic system in comparison to the conventional oil bath heating method [45,46]. To the best of our knowledge, this is the first attempt to use oxidovanadium(V) complexes immobilized on carbon supports for cyclohexane oxidation, under microwave conditions. The studied reaction is particularly important due to the significance of the oxidized products (KA oil, i.e., cyclohexanol (A, alcohol) and cyclohexanone (K, ketone)), in the manufacture of adipic acid, a relevant commodity, with a yearly production of over 3.5 million metric tons, growing ca. 5%/year [47] for the synthesis of nylon-6,6 polyamide. The current industrial homogeneous cyclohexane oxidation process bears serious limitations, as it can generate no more than 5–12% yield of KA oil to assure a reasonable selectivity of ca. 80–85% [48,49]. Thus, milder and more efficient alternatives are actively sought.

## 2. Materials and Methods

### 2.1. General Materials and Procedures

The synthetic part of this study was performed under atmospheric air using commercially available reagents and solvents, without further purification or drying. [VO(acac)_2_] was used as the metal source for the synthesis of the vanadium complexes.

C, H and N elemental analyses were carried out by the Microanalytical Service of the Instituto Superior Técnico (Lisbon, Portugal). Infrared spectra (4000–400 cm^−1^) were recorded on a Bruker Vertex 70 instrument (Bruker Corporation, Ettlingen, Germany) in KBr pellets, with wavenumbers in cm^−1^. The ^1^H and ^51^V NMR spectra were recorded at room temperature on a Bruker Advance II + 400.13 MHz (UltraShieldTM Magnet, Rheinstetten, Germany) spectrometer. Tetramethylsilane was used as the internal reference and the chemical shifts were reported in ppm. The catalytic tests performed under microwave (MW) irradiation were undertaken in a focused Anton Paar Monowave 300 microwave incorporating a rotational system and an IR temperature detector, using a 10 mL capacity reaction tube with a 13 mm internal diameter. Gas chromatographic (GC) measurements were carried out in a FISONS Instruments GC 8000 series gas chromatograph (Agilent Technologies, Santa Clara, CA, USA) with a capillary DB-WAX column (30 m × 0.32 mm) and a FID detector under the following conditions: program 120 °C for 1 min, 10 °C/min, 200 °C for 1 min, injector at 240 °C and helium as the carrier gas. Gas chromatography–mass spectrometry (GC–MS) analyses were performed using a Perkin Elmer Clarus 600 C (Shelton, CT, USA) instrument (He as the carrier gas). The ionization voltage was 70 eV. Gas chromatography was conducted in the temperature-programming mode, using a SGE BPX5 column (30 m × 0.25 mm × 0.25 m). All products achieved from the catalytic oxidation reactions were identified by their retention times, confirmed with those of commercially available samples. The mass spectra of reaction products were compared to fragmentation patterns obtained from the NIST spectral library stored in the computer software of the mass spectrometer.

#### 2.1.1. Synthesis of the Pro-Ligand H_2_L

**Scheme 1:**
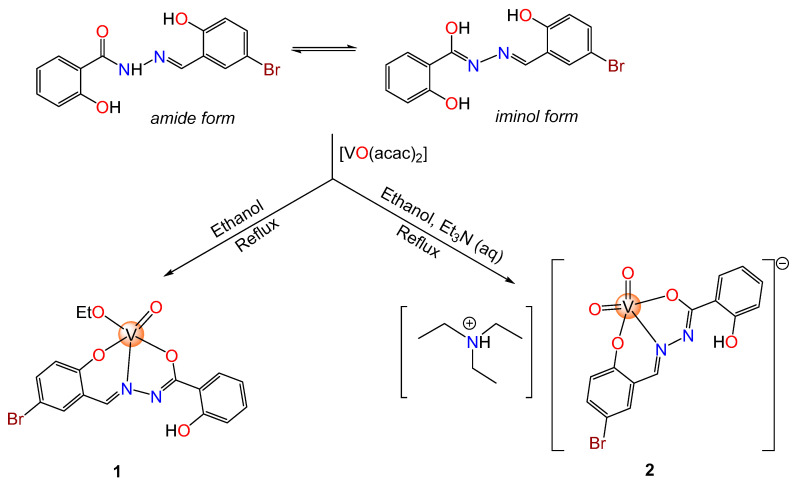
Syntheses of **1** and **2**.

The aroylhydrazone Schiff base pro-ligand (5-bromo-2-hydroxybenzylidene)-2-hydroxybenzohydrazide (H_2_L) (Scheme 1) was synthesized by a method similar to that reported earlier [36]. A total of 3.80 g (50 mmol) of 2-hydroxybenzohydrazide was dissolved in 50 mL ethanol and 5.02 g (25 mmol) 5-bromo-2-hydroxybenzaldehyde was added. The mixture was then refluxed for 2 h. The resulting yellow–white compound was then separated by filtration, washed thrice with cold ethanol and dried over fused CaCl_2_.

Yield: 86%. Anal. Calcd for C_14_H_11_BrN_2_O_3_: C, 50.17; H, 3.31; N, 8.36. Found: C, 50.12; H, 3.26; N, 8.30. 1H NMR (DMSO-*d*_6_, *δ*): 11.94 (s, 2H, OH), 11.20 (s, 1H, NH), 8.61 (s, 1H, −CH=N), 7.82–6.86 (m, 7H, C_6_H_4_). IR (KBr; cm^−1^): 3236 ν(OH), 3082 ν(NH), 1611 ν(C=O), 1556 ν(C=N). ESI-MS (+): *m/z* = 336.15 [M + H]^+^ (100%).

#### 2.1.2. Synthesis of the Oxidovanadium(V) Compound [VO(OEt)L] (1)

**Figure 1:**
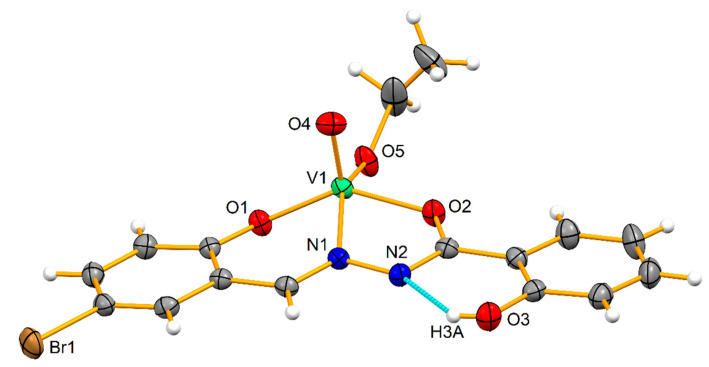
Ellipsoid plot of 1 (drawn at 30% probability level) with partial atom numbering scheme. Only one component of the disordered ethoxido group is represented. Selected bond distances (Å) and angles (°): N1–V1 2.118(3), O1–V1 1.834(3), O2–V1 1.932(3), O4–V1 1.585(3), O5–V1 1.753(3), N1–N2 1.391(4); O4–V1–O5 107.70(15), O4–V1–O1 107.64(15), O5–V1–O1 99.83(12), O4–V1–O2 104.23(14), O5–V1–O2 88.90(13), O1–V1–O2 142.38(12), O4–V1–N1 96.97(13), O5–V1–N1 152.97(14), O1–V1–N1 82.53(11), O2–V1–N1 74.18(11). Intramolecular D–H···A contact represented in dashed cyan color: O3···N2 2.615(4) Å, O3–H3A···N2 140(8)°.

A total of 0.265 g (1.00 mmol) of [VO(acac)_2_] was added to an ethanol suspension (30 mL) of H_2_L (0.335 g, 1.00 mmol), and the reaction mixture was refluxed for 2 h in an oil bath, in open air. The resultant reddish-brown solution was filtered, and the filtrate was kept in air for slow evaporation. After 3 days, crystals (Scheme 1 and Figure 1) were isolated from the solution, washed 3 times with cold ethanol and dried in open air.

Yield: 72%. Anal. calc. for **1** (C_16_H_14_BrN_2_O_5_V): C, 43.17; H, 3.17; N, 6.29. Found: C, 43.10; H, 3.13; N, 6.25. IR (KBr; cm^−1^): 3362 ν(OH), 1564 ν(C=N), 1254 ν(C–O)enolic, 1062 ν(N–N), 952 ν(V=O). ^1^H NMR (DMSO-*d*_6_, *δ*): 10.22 (s, 1H, OH), 8.92 (s, 1H, –CH=N), 7.84–6.72 (m, 7H, C_6_H_4_ and C_6_H_3_), 5.54 (q, 2H, CH_2_, OEt), 1. 64 (t, 3H, CH_3_, OEt). ^51^V NMR (DMSO-*d*_6_; *δ*) −548. ESI-MS (+): *m/z* 446 [**1** + H]^+^ (100%).

#### 2.1.3. Synthesis of the Dioxidovanadium(V) Compound [Et_3_NH][VO_2_L] (2)

[VO(acac)_2_] (0.265 g, 1.00 mmol) was added to an ethanol suspension (30 mL) of H_2_L (0.335 g, 1.00 mmol), and the reaction mixture was heated to reflux for 30 min in an oil bath in open air. A dark brown solution was obtained. Then, triethylamine (0.6 mL) and a few drops of water were added. The resultant yellow solution was then heated to reflux for another 30 min and filtered. The filtrate was left in air for slow evaporation. In ca. 3 days, X-ray-quality yellow single crystals were isolated, washed three times with cold ethanol and dried in open air.

Yield: 76%. Anal. calc. for C_20_H_25_BrN_3_O_5_V: C, 46.35; H, 4.86; N, 8.11. Found: C, 46.29; H, 4.80; N, 8.07. IR (KBr; cm^−1^): 3416 ν(OH), 1582 ν(C=N), 1256 ν(C–O)enolic, 1059 ν(N–N), 939, 908 ν(V=O). ^1^H NMR (DMSO-*d*_6_, *δ*): 12.10 (s, 1H, OH), 9.06 (s, 1H, –CH=N), 7.86–6.78 (m, 6H, C_6_H_4_), 3.09 (q, 6H, CH_2_, Et_3_NH), 1.20 (t, 9H, CH_3_, Et_3_NH). ^51^V NMR (DMSO-*d*_6_; *δ*) −536. ESI-MS(−): *m/z* 418 [VO_2_L]^−^ (100%).

### 2.2. X-ray Measurements

Single crystals of **1** and **2**, suitable for X-ray diffraction, were immersed in cryo-oil, mounted on Nylon loops and measured at 33 °C. Intensity data were collected using a Bruker APEX-II PHOTON 100 diffractometer with graphite mono chromated Mo-Kα (*λ* = 0.71073) radiation. Data were collected using phi and omega scans of 0.5° per frame and a full sphere of data was obtained. Cell parameters were retrieved using Bruker SMART [50] software and refined using Bruker SAINT [51] on all the observed reflections. Absorption corrections were applied using SADABS [51]. Structures were solved by direct methods by using SHELX-97 [52] and refined with SHELXL-2018/3 [53]. Calculations were performed using WinGX version 2014.1 [54]. All non-hydrogen atoms were anisotropically refined. The H-atoms bonded to carbon were included in the model at geometrically calculated positions and refined using a riding model, with U_iso_ defined as 1.2U_eq_ (for the aromatic and methylene groups) or 1.5U_eq_ (for the methyl groups) of the parent carbon atoms. The N- and O-bound hydrogen atoms were also included in calculated positions and refined. Least square refinements with anisotropic thermal motion parameters for all the non-hydrogen atoms and isotropic for the atoms were employed.

### 2.3. Carbon Materials Production and Treatments

Two types of carbon materials, already used in a previous work [13], were utilized as supports: activated carbon (AC) from Sigma–Aldrich Norit RO 0.8 and carbon nanotubes (CNT) from Nanocyl NC3100. These materials were tested in their original forms (AC and CNT), oxidized with nitric acid (5 M) under reflux for 3h (-ox) and oxidized with nitric acid and subsequently with NaOH (-ox-Na). In the last case, the -ox materials were further treated with NaOH (20 mM solution) for 1h, then washed and dried, as described in previous publications [13,14,15,16]. Six different carbon samples were obtained.

### 2.4. Carbon Materials Characterization

The carbon materials were previously characterized by N_2_ adsorption at −196 °C and by temperature-programmed desorption (TPD), as described in previous publications [13,14,15,16]. N_2_ adsorption isotherms were obtained in a Quantachrome Nova 4200e apparatus (Boynton Beach, FL, USA), using multipoint BET theory for specific surface area determination, the total pore volume was determined at the relative pressure of 0.99 and Boer’s t-method was used to determine the micropore volume. TPD experiments were carried out in an Altamira AMI-300 apparatus (Pittsburgh, PA, USA) with a coupled Ametek Dycor Dymaxion quadrupole mass spectrometer (Pittsburgh, PA, USA). Samples were heated under He flow at 5 °C/min heating rate up to 1100 °C. Desorbed CO and CO_2_ were monitored by mass spectrometry.

### 2.5. Heterogenization Protocol

Each complex was heterogenized on the 6 different carbon supports through dissolution in absolute ethanol with continuous stirring for 72 h, in an attempt to achieve 2 wt % (*p*/*p*) of V catalyst per gram of carbon. Afterwards, the material was collected by filtration, washed with ethanol and dried overnight at 40 °C, under vacuum.

### 2.6. Characterization of Heterogenized Complexes

The morphological characterization of the samples and the dispersion of the metallic particles were carried out by Field Emission Gun Scanning Electron Microscopy (FEG-SEM) with an X-ray Energy-Dispersive System (EDS) in a JEOL JSM-7001F (using an accelerating voltage of 25 kV, Tokyo, Japan) equipment, at Microlab, IST. The loading of V on the carbon materials before and after catalysis was determined by inductively coupled plasma-atomic emission spectrometry (ICP-AES) at the ReQuimTe Laboratory of Analysis and at Laboratório de Análises, IST, using a Horiba Jobin–Yvon Ultima apparatus, equipped with a RF generator of 40.68 MHz, and a Czerny–Turner monochromator with 1.00 m (sequential).

### 2.7. Catalytic Studies

#### General Procedure for the Peroxidative Oxidation of Cyclohexane

The catalytic tests under focused microwave (MW) irradiation were performed in an Anton Paar Monowave 300 microwave reactor, fitted with a rotational system and an IR temperature detector, using sealed cylindrical Pyrex tubes (10 mL capacity reaction vial with a 13 mm internal diameter). The peroxidative oxidation of cyclohexane (CyH) was performed using the following procedure: the catalyst, **1** or **2**, in homogeneous conditions or supported in the carbon materials (0.2 mol% vs. CyH), was dissolved in MeCN (3.00 mL), under vigorous stirring, followed by addition of CyH (5.00 mmol) and 70% aq. TBHP (10.00 mmol). The tube was placed in the MW reactor and the mixture was stirred (600 rpm) and irradiated (20 W) for 2 h at 80 °C. After the reaction, the mixture was cooled to room temperature and the suspension (in case a supported catalyst was used) was centrifuged and filtrated to prepare the samples for GC analysis (internal standard method) using nitromethane (50 μL) as standard compound. Prior to the GC analysis, an excess of triphenylphosphine was added (to reduce the formed cyclohexyl hydroperoxide to the corresponding alcohol, following a method developed by Shul’pin [55,56,57,58]). All the catalytic assays under MW irradiation were performed in duplicate, to assure reproducibility of the results. Blank experiments (without a catalyst) were performed and confirmed that no product of cyclohexane oxidation was detected in a significant amount. V leaching to the reaction solution for the best-performing catalysts was investigated by ICP-AES, using the conditions described in 2.6.

Recyclability of the best-performing catalysts was investigated. After completion of each run, the products were separated for analysis as above-mentioned and the catalyst was recovered by filtration, thoroughly washed with acetonitrile and dried overnight in an oven at 50 °C. Each new catalytic cycle was initiated after the preceding one, upon addition of new portions of reagents. The recycling tests were carried out for up to four consecutive cycles.

Chromatographic analyses were undertaken by using a Clarus 500 GC series gas chromatograph with a DB-624 (J&W) capillary column (DB-WAX, column length: 30 m; internal diameter: 0.32 mm), flame ionization detector (FID) and the Total Chrom software. The injection temperature was 240 °C. After the injection, the reaction temperature was maintained at 100 °C for 1 min, then increased at 10 °C/min to 160 °C and held at this temperature for 1 min. Helium was used as the carrier gas. The products were identified by comparison of their retention times with those of known reference compounds.

GC–MS analyses were performed by using a PerkinElmer Clarus 600 C instrument (with He as the carrier gas). The ionization voltage was 70 eV. GC was conducted in the temperature-programming mode using an SGE BPX5 column (30 m × 0.25 mm × 0.25 mm). Reaction products were identified by comparison of their retention times with those of known reference compounds, and by comparing their mass spectra to fragmentation patterns obtained from the NIST spectral library stored in the computer software of the mass spectrometer.

## 3. Results and Discussion

Aroylhydrazone (5-bromo-2-hydroxybenzylidene)-2-hydroxybenzohydrazide was used to synthesize the oxidovanadium(V) compound [VO(OEt)L] (**1**) and the dioxidovanadium(V) complex [Et_3_NH][VO_2_L] (**2**) (Scheme 1). In air, the reaction of H_2_L with [VO(acac)_2_], in refluxing ethanol, leads to the formation of the oxidoethoxidovanadium(V) complex **1**. The presence of aqueous triethylamine in the above reaction mixture originates the (salt-like) dioxidovanadium(V) compound **2**. In both cases, the ligand exhibits the *iminol (enol)* form commonly found in this type of aroylhydrazone ligand [6,13,35,37,59,60,61,62,63] and acts as a di-negative chelator L^2−^.

Complexes **1** and **2** were characterized by elemental analysis, infrared spectroscopy (IR), ^1^H and ^51^V nuclear magnetic resonance (NMR) spectroscopy, electrospray ionization mass spectrometry (ESI-MS) and X-ray diffraction (single crystal) analyses. The IR spectra shows the similar characteristic stretching signals of the coordinated tridentate anionic ligand L^2−^ in the enolate form (see Materials and Methods), along with the typical ν(V=O) bands in the 908–952 cm^−1^ region [4,13,35,36,37,38,44]. In the case of the dioxidovanadium(V) complex **2**, the two ν(V=O) modes correspond to the symmetric and antisymmetric pairs of *cis* V=O groups [4,13,35,44,59,60,61,62].

The ^1^H NMR spectra of the pro-ligand H_2_L, **1** and **2** were obtained in DMSO-*d*_6_. Complexes **1** and **2** exhibit all the characteristic resonances of the ligand [34]. In addition, for **1**, the methylene and methyl protons of the ethoxido ligand resonate as the quartet and triplet at *δ* 5.54 and 1.64, respectively. In the case of **2**, the presence of the triethylammonium ion is also observed in the ^1^H NMR spectrum as a quartet and triplet at *δ* 3.09 and 1.20, respectively. In the ^51^V NMR spectra, a peak at −548 or −536 ppm was found for **1** or **2**, respectively, being typical of oxidovanadium(V) centers [5,13].

The ESI-MS spectra of **1** and **2** display the corresponding molecular ion peaks (in the case of **2** only the complex moiety), which also support the formulations.

### 3.1. X-ray Structures

X-ray-quality single crystals of **1** and **2** were obtained from ethanol. The crystallographic data and processing parameters are summarized in Table 1.

**Figure 2:**
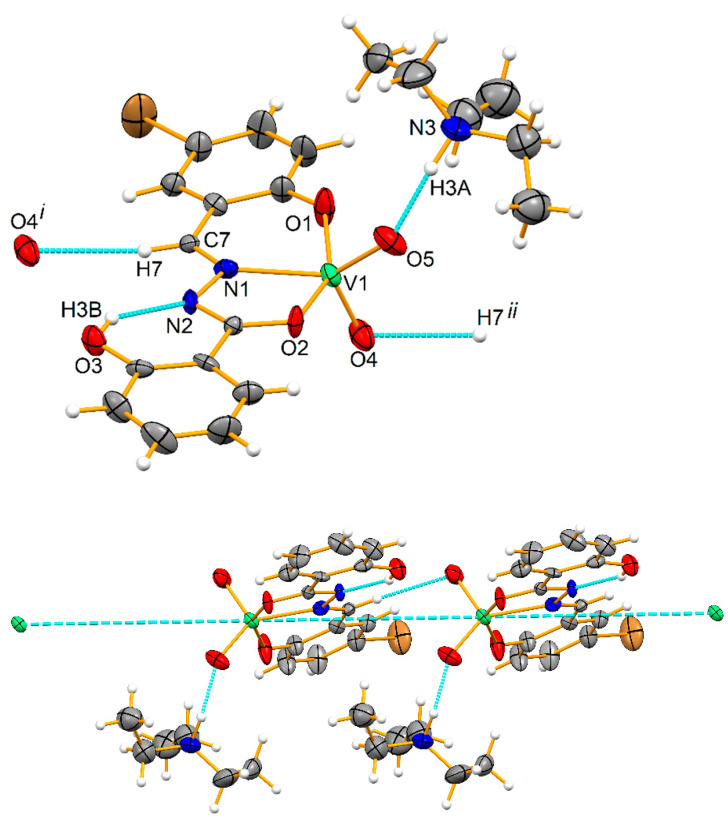
Ellipsoid plot of **2** (drawn at 30% probability level) with partial atom numbering scheme (**top**) and a fragment of the molecules alignment along the *b* axis (**bottom**). Only one component of the disordered triethylammonium cation is represented. Selected bond distances (Å) and angles (°): N1–V1 2.149(12), O1–V1 1.925(11), O2–V1 1.997(9), O4–V1 1.599(13), O5–V1 1.644(12), N1–N2 1.419(14); O4–V1–O5 107.3(7), O4–V1–O1 103.9(7), O5–V1–O1 95.6(6), O4–V1–O2 100.0(6), O5–V1–O2 93.2(5), O1–V1–O2 150.7(5), O4–V1–N1 113.9(5), O5–V1–N1 138.3(6), O1–V1–N1 80.8(4), O2–V1–N1 74.1(4). Intra- and intermolecular D–H⋅⋅⋅A contacts represented in dashed cyan color: O3⋅⋅⋅N2 2.566(15) Å, O3–H3B⋅⋅⋅N2 144°; N3⋅⋅⋅O5 2.70(2) Å, N3–H3A⋅⋅⋅O5 156°; C7⋅⋅⋅O4 3.402(17) Å, C7–H7⋅⋅⋅O4 158°. Symmetry operations to generate equivalent atoms: (*i*) x, 1 + y, z, (*ii*) x, −1 + y, z.

The asymmetric unit of **1** contains an oxidovanadium(V) (VO^3+^) cation, an L^2−^ and an ethoxido (EtO^−^) ligand (Figure 1), while **2** involves the hydrazone L^2−^ ligand on a dioxidovanadium(V) VO_2_^+^ center and a triethyl ammonium cation (Figure 2). The vanadium atoms display square pyramidal environments (τ = 0.18 (**1**) and 0.21 (**2**)) [63], in which the basal planes are made up from the O_phenolate_, the O_enolate_ and the N_imine_ from the L^2−^ ligands and an O_ethoxido_ (in **1**) or O_oxido_ (in **2**); the axial positions are engaged with an O_oxido_ in both cases. Resulting from the chelation nature of L^2−^, the vanadium cations are involved in six- and five-membered rings with bite angles of 80.8(5)° and 74.0(4)° for **1**, or of 82.5(1)° and 74.2(1)° for **2**, respectively, which are similar to those already reported for other oxidovanadium(V) compounds [4,13,59,60,61,62]. The vanadium atoms are displaced, relative to the least-square planes defined by the *NO*_3_ coordinating atoms, towards the axial ligands by 0.476 (**1**) and 0.516 Å (**2**). Moreover, in both compounds, the bromophenyl moieties stand on those planes but the phenolates are clearly displaced, with their mean planes making angles of 7.27° (**1**) and 17.93° (**2**) with the *NO*_3_ coordinating one.

The bond lengths and angles of **1** and **2** are similar (see captions of Figure 1 and Figure 2). The vanadium–oxygen bond lengths follow the order: V−O_oxido_ < V−O_ethoxido_ < V−O_phenoxido_ < O_enolate_, commonly observed in this type of complex [4,13,35,36,37].

The minimum V⋅⋅⋅V distance in **2** (7.141(3) Å, coincident with the unit cell *b* dimension) is longer than the value found in **1** (6.321(1) Å), which is conceivably related with the orientation of the VO_2_ groups against the plane of the L^2−^ ligand in vicinal molecules (Figure 2, bottom), positioning them in line along the crystallographic *b* axis. 

Intramolecular H-bond interactions are found in **1** and **2**, involving the aromatic hydroxyl and the non-coordinated N_azo_-atoms (Figure 1 and Figure 2) in typical $S_{1}^{1}\left(6\right)$ graph sets. The triethylammonium protons and the O_oxido_ atoms in **2** are in contact through $D_{1}^{1}\left(2\right)$ intermolecular interactions.

### 3.2. Characterization of Carbon Supports and Heterogenized Materials

**Figure 3:**
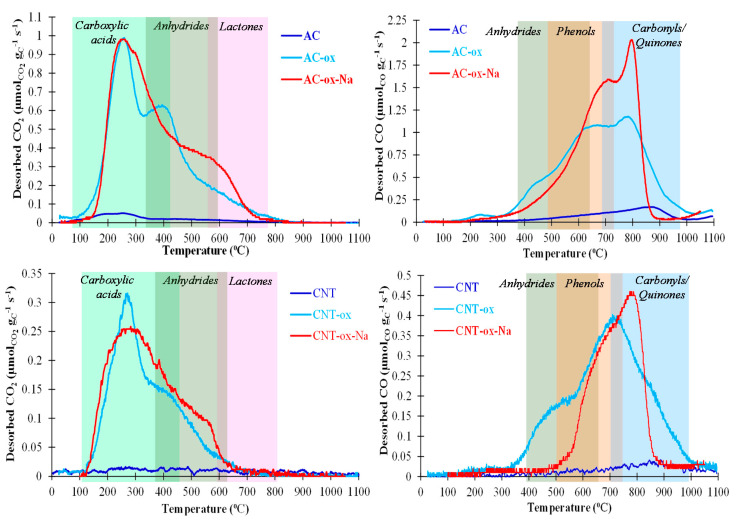
TPD profiles for AC (**top**) and CNT (**bottom**). The desorption of CO_2_ (**left**) and CO (**right**) is shown with the identification (in color bars) of the types of groups that desorb at different temperature ranges (adapted from [13]).

The carbon supports were prepared and characterized in a previous work [13]. They were used as purchased (AC and CNT), oxidized with nitric acid (-ox) and oxidized with nitric acid and subsequently treated with sodium hydroxide (-ox-Na), as referred to above. A resume of the previously performed N_2_ adsorption and TPD results can be found in Table 2. The corresponding N_2_ adsorption isotherms can be found in Supporting Information (Figures S1 and S2) and TPD graphics in Figure 3.

It was found that AC has a rich surface chemistry, characterized by a large amount of surface oxygenated groups, desorbed as CO and CO_2_ (Figure 3 and Table 2), and also the largest surface area and micropore volume (Table 2). In fact, this material is mostly microporous, as shown by the characteristic type I isotherm (Figure S1), with type IV hystheresis, typical of slit-like pores [64]. The CNTs have a lower surface area (Table 2) due to their cylindrical structure and many less surface groups (Figure 3 and Table 2). CNT shows a type IV isotherm with type H1 hystheresis (Figure S2), characteristic of materials with cylindrical pore geometry and a high degree of pore size uniformity [64].

Upon oxidation treatment (-ox), the surface area and porosity of AC-ox slightly decreased, compared to AC (Table 2), due to the pore wall collapse caused by the acid treatment, which might partially block the access of N_2_ molecules to the micropores; however, not many differences are seen in the isotherms (Figure S1). Since nanotubes are generally closed at either end, the use of a strong oxidizing agent such as nitric acid can open the nanotube tips, explaining the increases in surface area for CNT-ox, compared to CNT (Table 2). The hystheresis resembles more a H2 type, characteristic of a larger presence of mesopores, compared to pristine CNT (Figure S2). Additionally, the amounts of surface groups desorbed, and CO and CO_2_ in the TPD, increase for both types of materials (Figure 3 and Table 2). This is mainly due to the creation of carboxylic acid groups on the support surface, but also of anhydrides and phenols (Figure 3) [13].

Upon further treatment with NaOH (-ox-Na), the surface groups are converted to (sodium) carboxylates, that are more stable, and also cause a slightly larger signal in TPD (Figure 3 and Table 2) [13]. A larger pore wall collapse might cause a further decrease in the surface area in AC-ox-Na. In terms of CNT-ox-Na, the NaOH treatment could have blocked those previously opened tips (of CNT-ox), decreasing the surface area. Not many changes are seen in the isotherms (Figures S1 and S2). In terms of the porosity values, CNT have a cylindrical structure, as referred to above, and the pores result from the free space in the bundles. The presence of surface groups created by the treatments can also modify this space, explaining the different values obtained for the CNT-ox and CNT-ox-Na samples.

**Figure 4:**
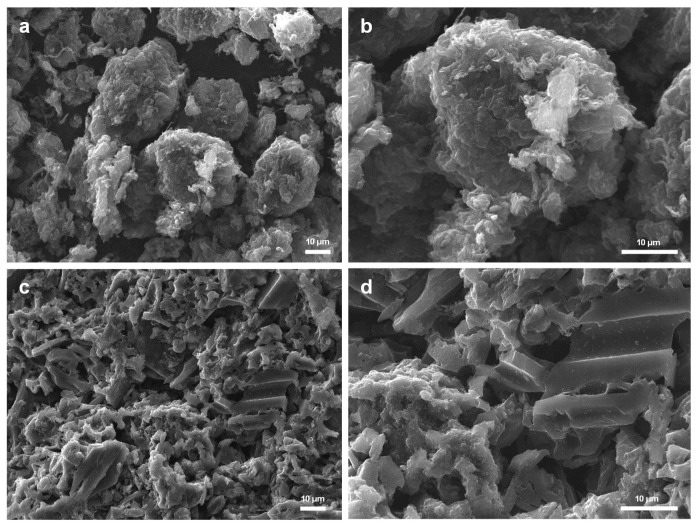
SEM images of the heterogenized samples: (**a**,**b**) **1**@CNT-ox; (**c**,**d**) **2**@AC-ox.

The morphology of the carbon materials with the anchored complexes and the distribution of the metallic species were also examined by SEM for two representative samples (Figure 4, Figures S3 and S4). The SEM images of the heterogenized materials resemble the morphology of the pristine carbon supports (not shown). A clear difference is observed between the morphology of the two different carbon supports. For CNT, a more ordered structure is observed, with similar aggregates, whereas AC presents a more disordered structure, as expected, with high porosity (it is a commercial material obtained by steam activation of peat). A homogeneous distribution of vanadium particles on the selected carbon supports was observed by the EDS mapping (Figures S3 and S4).

### 3.3. Heterogenization Efficiency

Aiming at achieving catalyst recycling and/or enhancement of the catalytic properties of the oxidovanadium(V) complexes, **1** and **2** were heterogenized on the different carbon materials, in their original forms (AC and CNT), oxidized with HNO_3_ (AC-ox and CNT-ox) or oxidized with HNO_3_ followed by refluxing with NaOH (AC-ox-Na and CNT-ox-Na). The establishment of a covalent bond between the metal center and surface groups of the carbon supports is assumed, this being a well-known strategy to immobilize transition–metal complexes with catalytic properties [65], although non-covalent interactions (e.g., hydrogen bonds) can also play a role. The intention was to heterogenize ca. 2% of vanadium, but different loadings were obtained on the complexes by ICP-AES, as displayed in Table 3.

As seen above, although the porous structure and surface chemistry of the supports are affected by the different treatments, all the obtained materials were able to anchor the V complexes, although with different efficiencies. However, the vanadium compounds, in general, heterogenize better on -ox samples than on ox-Na supports, unlike what was observed in previous works [13]. Particularly, the immobilization of both complexes seems to be more favorable in CNT-ox than in CNT-ox-Na support. A possible explanation is as follows: the V complex in **2** is anionic and thus conceivably can interact better with a neutral functional site of the C-ox support (e.g., a phenol or carboxylic acid) than with an ionic site (phenolate or carboxylate, respectively). For **1**, the possible reason is not so straightforward, but may be due to the lability of the EtO^−^ ligand upon reaction with a protic reagent (such as a phenol or a carboxylic acid) to form labile EtOH that is liberated [66,67], allowing the ligation of the V to the support.

In addition, complex **2** heterogenizes better on AC samples than on CNT materials, while for **1**, the opposite is observed. A better heterogenization on AC is common, since this material has a large surface area and a very rich surface chemistry itself (Table 2), thus providing a good environment for the anchorage of complexes.

### 3.4. Microwave-Assisted Oxidation of Cyclohexane 

**Scheme 2:**
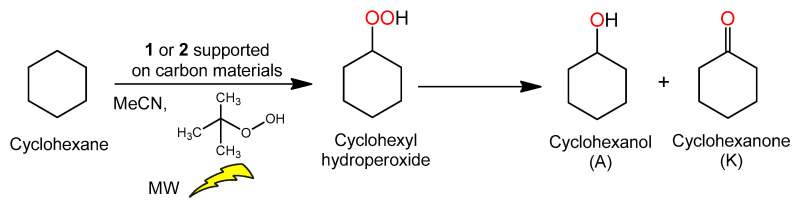
MW-assisted oxidation of cyclohexane to cyclohexyl hydroperoxide, cyclohexanol and cyclohexanone with aqueous TBHP catalyzed by **1** and **2** supported on the different carbon materials.

Cyclohexane was used as a model substrate to investigate the catalytic activity of the oxovanadium complexes. The catalytic systems for the oxidation of cyclohexane were based on the vanadium complexes **1** and **2** supported on the different carbon supports with different surface chemistries, consisting of the MW-assisted peroxidative oxidations performed under mild conditions, using *tert*-butyl hydroperoxide (TBHP, 70% aq. solution) as oxidant and acetonitrile (MeCN) as solvent. The choice of the solvent was made owing to its high resistance to oxidizing agents and also to the good solubility of the substrate. The catalytic performance of the hybrid materials was evaluated in terms of product yield, turnover number (TON) or frequency (TOF, h^−1^) and selectivity as a function of the carbon support (Table 4). MW irradiation was used as an alternative energy source to the conventional heating method. This source of energy proved to significatively enhance the catalytic activity of these systems and promote product yield [68,69]. Complexes **1** and **2** were able to catalyze the MW-assisted oxidation of cyclohexane to a mixture of cyclohexanone and cyclohexanol (the so-called KA oil) using TBHP as the oxidizing agent, under low-power (20 W) irradiation, both in homogeneous phase and heterogenized on the carbon materials (Scheme 2). Cyclohexyl hydroxyperoxide is the primary product of the conversion of cyclohexane, that is subsequently converted to the alcohol and ketone.

### 3.5. Catalytic Tests for the Homogeneous Catalysts

The oxidovanadium complexes **1** and **2** were tested as catalysts for the homogeneous MW-assisted cyclohexane oxidation reaction, with TBHP as oxidant and MeCN as solvent, to cyclohexanone and cyclohexanol. Under the optimized conditions for these additive-free systems (80 °C and 2 h of low power (20 W) MW irradiation), in homogeneous conditions, yields of KA oil of 6.1 and 7.4% (for complex **1** and **2**, respectively) were obtained, using a low vanadium catalyst load (0.2% molar ratio, relative to cyclohexane). These yields are comparable to those reported for the industrial process (8%), although lower than previously reported values [35]. A high selectivity was observed towards the formation of KA oil with these two complexes, as cyclohexanol and cyclohexanone were the only products detected by GC–MS analysis, and under these assayed conditions, no further oxidation products were detected, thus revealing a very selective oxidation system.

### 3.6. Catalytic Tests for the Heterogenized Catalysts

**Figure 5:**
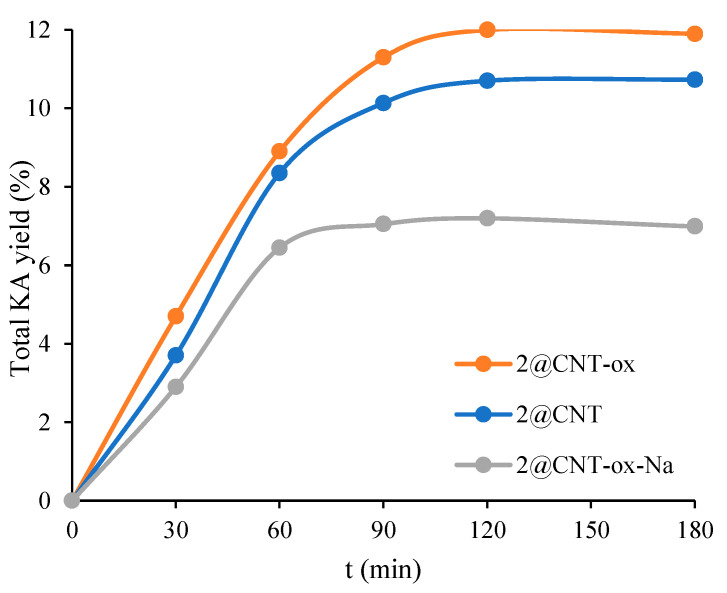
Effect of the duration of the MW irradiation on the catalytic activity of 2@CNT materials for the oxidation of cyclohexane with TBHP in MeCN.

The compounds **1** and **2** heterogenized on the carbon materials were successfully tested towards the MW-assisted cyclohexane oxidation reaction. The catalytic reactions were monitored over time to study the influence of the MW irradiation time on the catalytic performance of the hybrid materials, namely on the yield of KA oil (Figure 5 and Figure S6). An increase in the reaction time from 0.5 to 2 h enhanced the KA oil yields. After this irradiation time, under the above conditions, the yield augment was not significant, so 2 h was chosen as the optimal reaction time.

The catalytic activity of the supported materials was dependent on the support (Figure S5 and Table 4), but the selectivity was maintained (no traces of by-products were detected by GC–MS analysis). The carbon supports alone, on their original forms (AC, CNT), oxidized with HNO_3_ (-ox) or oxidized with HNO_3_ followed by refluxing with NaOH (-ox-Na) were also tested for cyclohexane oxidation, under the same conditions, but showed no catalytic activity.

Cyclohexyl hydroperoxide (CyOOH) is formed as a primary product and evolves in a mixture to the final products, cyclohexanol and cyclohexanone (Scheme 2). The formation of CyOOH was proved by using Shul’pin’s method [55,56,57,58] (see Materials and Methods): prior to the GC analysis, the addition of PPh_3_ of the products resulted in an increase in the amount of cyclohexanol (due to the reduction of CyOOH by PPh_3_ and formation of phosphine oxide) and in a decrease in the amount of cyclohexanone, relative to the values obtained for the sample not subjected to reduction. The product yields given in Table 4 were obtained upon addition of PPh_3_.

The observed catalytic activity of the carbon-supported materials was higher than the values obtained for their homogeneous counterparts, except in the case of pristine AC. Regarding the AC systems, the AC-ox-Na support showed the best results in terms of KA oil yield, with totals of 7.2 and 10.3%, respectively, for supported **1** and **2**. On the other hand, for the CNT series, the CNT-ox systems were the most active, both for complex **1** and **2**, for the same V loading and reaction time. Overall, the most active heterogeneous system was **2**@CNT-ox (Figure S6 and Table 4, entry 13) with a maximum yield of KA oil of 12.0%. A high selectivity (>98%) towards the formation of KA oil was exhibited by the studied systems (no traces of by-products were detected by GC–MS analysis of the final reaction mixtures).

Although it has been previously shown that CNT-ox-Na is usually the best combination of support and treatment for catalytic activity and recycling ability [13,14,15,16], this was not found in this work. As shown in the heterogenization efficiency (Table 3), the ox-Na treatment did not improve the % of V anchored on the supported materials. The best results obtained for CNT, compared to AC, may be explained by the textural properties of this material, namely, its mesoporosity, that facilitates the access of the reactants to the active sites and also the electronic effects, attributed to enhanced interactions of its graphitic structure with the reactants or intermediates. The lower activities observed for the AC materials could result from a tighter entrapment of the immobilized complexes, as a consequence of a compulsory conformation, and to reduced or even blocked mass transport in the narrow (micro)porosity, imposing limits on the effective range of substrates that can be used.

Under the tested conditions, the peroxidative oxidation cyclohexane is believed to proceed mainly through a radical mechanism, similar to that proposed in other cases [13,35]. The reaction is carried out through formation of the cyclohexyl hydroperoxide (primary product), according to Scheme 2.

Possible leaching of the active metal species and the heterogeneous character of the system were tested by ICP-AES analysis of vanadium on the solution media and also by a hot filtration test, consisting of the removal of the catalyst from the reaction medium by filtration after 1 h of MW-assisted reaction of the best performing system, and subsequent continuation of the MW-irradiation of the solution for one more hour of reaction. Regarding the ICP-AES analysis to determine the amount of vanadium in the best-performing system, the **2**@CNT-ox, a 37% V leaching was detected in the solution media after the completion of the 2 h of MW-assisted reaction. However, the analysis of the final solution, obtained when performing the hot filtration test, revealed no increase in the oxygenated products of cyclohexane. This suggests that although vanadium is detected in solution, the vanadium species detached from the support to the solution were not catalytically active [70]. The presence of a significant remaining concentration of TBHP in solution was confirmed by GC, therefore assuring that enough oxidant was still in solution and that conversion of cyclohexane could be possible if the vanadium species in solution were able to catalyze the reaction.

The comparison of the obtained data with those reported in the literature is not straightforward, as very few reports can be found on carbon heterogenized catalysts based on oxidovanadium(V) complexes for the catalytic cyclohexane oxidation reaction. Table S1 provides an overview of the reported carbon-supported oxidovanadium complexes used as heterogeneous catalysts in the last decade. These materials were mostly used in selective oxidation reactions, with moderate to good results being obtained. The most reported carbon supports for these vanadium complexes are modified graphene oxide [28,31,32,34], CNT [13,26,29] and CMK-3 mesoporous carbon [30,33]. The oxidation of 1-phenylethanol and benzhydrol afforded 96% yield of acetophenone and benzohydrazone, using functionalized CNT [13] and graphene [28], respectively, with excellent selectivities. Regarding cyclo-octene oxidation with hydroxyl-functionalized oxovanadium(IV) Schiff-base supported onto modified multi-walled carbon nanotubes (MWCNT), a mixture of three oxidation products was obtained: cyclooctene epoxide (18.6%), cyclooctene-1-ol (52.5%) and cyclooctene-1-one (28.9%) at 83.4% conversion. Styrene oxidation has been, so far, the reaction with more reported heterogeneous catalysts based on oxovanadium complexes supported on carbon materials. Higher yields (67 and 56%) and selectivities (71 and 59%) were obtained for styrene oxide and epoxide when using amino-modified CMK-3 [30] and carbon-coated Fe_3_O_4_ hybridized with graphene [25], respectively, when compared to graphene oxide [31,34]. Verma et al. reported a conversion of 98% for oleic acid with 99% selectivity for the correspondent epoxide, using graphene oxide as support [32]. The epoxidation of geraniol with oxovanadium(IV) acetylacetonate supported onto CMK-3 has led to excellent results, with conversions > 98% and 99% selectivity for 2,3-epoxygeraniol. Amino-functional microporous organic nanotube frameworks were used as supports of an oxovanadium (IV) complex for the oxidation of *p*-chlorobenzenethiol, reaching 100% conversion with 98% selectivity. All the works gathered in Table S1 address catalyst stability, reporting the reuse of the materials for at least two cycles. Overall, the loss of catalytic activity lies within 5–10% of the initial activity and selectivity. Nevertheless, the gathered data on this topic are still insufficient when compared to the use of other materials as supports for vanadium complexes.

Regarding the reaction in study in this work, Table S2 shows representative studies on the use of supported vanadium complexes for the oxidation of cyclohexane. A comparison of our results is not straightforward, since apart from the present study, only one more report was found in the literature, focused on the use of oxidovanadium complexes supported on carbon materials for cyclohexane oxidation [27]. The remaining examples correspond to the use of other types of supports, namely zeolites and silicas. Regardless of the difference in the kind of support used, our catalytic system, even though presenting moderate yields (12%), compares favorably in terms of selectivity to KA oil with others reported in the literature [27,71,72]. Similar selective systems to KA oil were reported by Santra et al. and Ottaviani and colleagues [73,74], using silicas and zeolite as supports for vanadium phosphorus oxide and a V–scorpionate complex, respectively, although presenting a higher selectivity to cyclohexanone. Moderate to good yields of KA oil were obtained in these cases (30–50%). Salavati-Niasari et al. reported the use of functionalized oxidovanadium(IV) Schiff base immobilized onto modified MWCNTs, obtaining 78% conversion and a mixture of cyclohexanol, cyclohexanone and cyclohexane-diol, with a selectivity of 90.5% to KA oil [27]. The use of SBA-15 and TUD-1 silicas for supporting vanadium phosphorus oxide appears to be more advantageous for obtaining a 100% selectivity to KA oil [73], when compared to vanadium phosphate supported onto mesoporous KIT-6 silica, that afforded a 70% selectivity to KA oil [71]. The visible light-induced oxidation of cyclohexane by sulfated vanadium-doped zeolite TS-1, on its turn, supplied a different distribution of the oxidation products, with 0.1% of cyclohexanol, 2.7% of cyclohexanone and 13.4% of chlorocyclohexane.

### 3.7. Recycling

**Figure 6:**
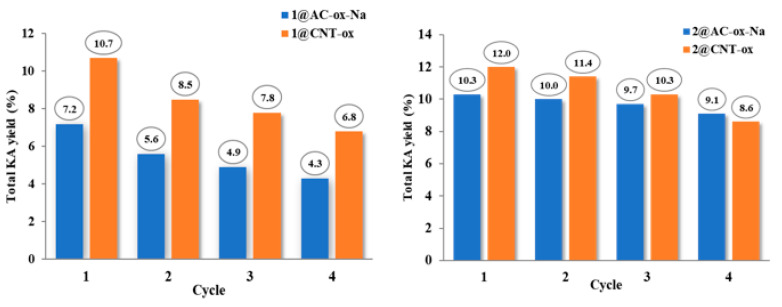
Recyclability of **1**@AC-ox-Na and **1**@CNT-ox (**left**) and of **2**@AC-ox-Na and **2**@CNT-ox (**right**) catalysts in the oxidation of cyclohexane to yield KA oil.

Under homogeneous conditions, the performance of consecutive catalytic cycles is not possible since the separation of the catalytic V species is not feasible. The stability of the most active catalytic systems, for both complexes, using @AC-ox-Na and @CNT-ox as supports was evaluated, by studying the recyclability of the hybrid materials in four consecutive catalytic cycles. The results are depicted in Figure 6.

In the case of complex **1**, a significant difference can be observed between the two studied systems in terms of the total KA oil yield. The use of AC-ox-Na as a support for **1** leads to a drop of nearly 40% in the yield of cyclohexane and cyclohexanol after the 4th cycle, whereas for complex **2**, the catalytic activity drops only 12% by the 4th cycle.

The difference observed in terms of total yield for the two supports is smaller for complex **2**, and the obtained results are very close for both supports. In fact, after the 4th cycle, **2**@CNT-ox maintains 72% of its initial activity, while **2**@AC-ox-Na preserves 88% of the activity found in the first cycle. Overall, the heterogenization of **2** seems to be more effective. Thus, by the 4th catalytic cycle, the systems **2**@AC-ox-Na and **2**@CNT-ox still show high KA oil yields. These materials can thus be competitively reused in more cycles. V leaching into the solution at the end of the recycling experiments was assessed for the spent catalyst **2**@CNT-ox. ICP-AES analysis of the recovered solid revealed that by the 4th cycle, 52% of the initial V content of this catalyst was detached from the carbon support. When comparing this value with that found after the first run, 37%, it can be concluded that the main drop in V load occurred after the first cycle. A smaller contribution to the decrease in the V content of the catalysts most probably occurred in the following reuse cycles. Therefore, the loss of activity for the heterogenized materials is related to leaching of the vanadium species after successive runs, but it should also be related to some blockage of the catalytic sites on the surface of the porous materials, especially in the case of activated carbons, due to their microporosity that limits the accessibility of the species to the inner pore network [75]. The stability of the described catalysts is in line with that described in the majority of the examples gathered in Table S2, that account for similar decreases in activity in recycling catalytic cycles and metal leaching [24,71,73]. Other studies on this topic did not address catalyst stability issues [72,73].

## 4. Conclusions

This work aims at contributing to the research in the field of heterogenization of vanadium complexes on carbon supports, a still underdeveloped area [1]. Oxidovanadium(V) and dioxidovanadium(V) compounds were anchored on functionalized carbon nanotubes and activated carbon and the obtained materials were tested on the solvent-free peroxidative oxidation (with TBHP) of cyclohexane. The immobilization of **1** and **2** resulted in an increase in the catalyst efficiency and allowed catalyst recyclability for up to four consecutive cycles, although with some loss of activity, more noticeable for heterogenized complex **1** than for complex **2**. Nevertheless, heterogenization allowed the reuse of these materials in several cycles, which was not possible for the homogenous analogues.

We trust this work will open a plethora of future applications for new immobilized vanadium (and other metal) complexes, for the oxidation of different alkanes (apart from cyclohexane) and other catalytic reactions, using MW irradiation.

## Figures and Tables

**Table 1:** Crystal data and structure refinement details for **1** and **2**.

	1	2
Empirical formula	C_16_H_14_BrN_2_O_5_V	C_20_H_25_BrN_3_O_5_V
Formula weight	445.14	518.28
Crystal system	Triclinic	Orthorhombic
Space group	*P*-1	*Pna*2_1_
*a*/Å	7.5794(15)	35.988(3)
*b*/Å	8.6682(17)	7.1406(6)
*c*/Å	13.170(3)	8.9154(8)
α/°	89.896(7)	90
*β/*°	80.633(8)	90
γ/°	80.921(7)	90
*V* (Å^3^)	842.8(3)	2291.0(3)
*Z*	2	4
D_calc_ (g cm^−3^)	1.754	1.503
F000	444	1056
*μ*(Mo K*α*) (mm^−1^)	2.989	2.212
Rfls. collected/unique/observed	14,774/3453/3025	26,703/4074/3415
*R*_int_	0.0727	0.0969
Nº parameters	250	310
Final *R*1 ^*a*^, *wR*2 ^*b*^ (*I* ≥ 2*σ*)	0.0657, 0.1779	0.0969, 0.2199
Goodness-of-fit on *F*^2^	1.029	1.157

*^a^* R *=* Σ||*F_o_*| − |*F_c_*||/Σ|*F_o_*|; ^*b*^ wR(*F*^2^) = [Σw(|F_o_|^2^ − |F_c_|^2^)^2^/Σw|F_o_|^4^]^½^.

**Table 2:** Characterization of carbon supports: surface area, total pore volume and micropore volume obtained by adsorption of N_2_ at −196 °C and amounts of CO and CO_2_ desorbed, determined by TPD (adapted from [13]).

Sample	Surface Area (m^2^/g)	Total Pore Volume (cm^3^/g)	Micropore Volume (cm^3^/g)	CO_2_ Desorbed (µmol/g)	CO Desorbed (µmol/g)
AC	974	0.67	0.348	179	643
AC-ox	914	0.62	0.324	2596	4930
AC-ox-Na	610	0.35	0.251	2883	5012
CNT	257	2.89	~0	89	142
CNT-ox	400	1.89	~0	729	1475
CNT-ox-Na	350	1.45	~0	838	1079

**Table 3:** Metal loading (% p/p) of V on the carbon materials determined by ICP-AES.

Samples	Metal (%, *p*/*p*)	
	1	2
AC	0.25	0.87
AC-ox	0.19	0.85
AC-ox-Na	1.12	0.65
CNT	1.51	0.53
CNT-ox	1.14	0.76
CNT-ox-Na	0.83	0.36

**Table 4:** MW-assisted catalytic oxidation of cyclohexane with TBHP in MeCN, catalyzed by complexes **1** and **2** supported on different carbon materials (selected data) *^a^*.

Entry.	Catalyst	TON *^b^*	TOF (h^−1^)	Yield (%) *^c^*		
				A	K	Total
**1**	1	61	31	3.0	3.1	6.1
**2**	1@AC	50	25	1.8	2.2	4.0
**3**	1@AC-ox	72	36	4.0	3.2	7.2
**4**	1@AC-ox-Na	68	34	3.2	3.6	6.8
**5**	1@CNT	77	39	4.0	3.7	7.7
**6**	1@CNT-ox	107	54	5.4	5.3	10.7
**7**	1@CNT-ox-Na	72	36	3.3	3.9	7.2
**8**	2	74	37	2.7	4.7	7.4
**9**	2@AC	40	20	2.4	2.6	5.0
**10**	2@AC-ox	83	42	5.0	3.3	8.3
**11**	2@AC-ox-Na	103	52	4.6	5.7	10.3
**12**	2@CNT	107	54	5.2	5.5	10.7
**13**	2@CNT-ox	120	60	7.0	5.0	12.0
**14**	2@CNT-ox-Na	72	36	3.7	3.1	7.2
**15**	-	6	3	0.4	0.2	0.6

*^a^* Reaction conditions: MW (20 W), acetonitrile (3.0 mL), cyclohexane (5.0 mmol), TBHP (70% aq. sol., 10.0 mmol), catalyst (5.0 µmol), 2.0 h, 80 °C. *^b^* Total (cyclohexanol + cyclohexanone) turnover number (moles of product per mol of supported catalyst) determined by GC analysis (upon treatment with PPh_3_). *^c^* Molar yields based on substrate determined by GC analysis (upon treatment with PPh_3_), i.e., moles of products (cyclohexanol+cyclohexanone) per 100 mol of cyclohexane.

## Data Availability

Data will be made available upon request.

## Supplementary Materials

The following are available online at https://www.mdpi.com/article/10.3390/nano11061456/s1, Figure S1: N_2_ adsorption isotherms of AC, AC-ox, AC-ox-Na, Figure S2: N_2_ adsorption isotherms of CNT, CNT-ox, CNT-ox-Na, Figure S3: SEM-EDX mapping for sample V1@CNT-ox: C (green), O (pink), Br (blue) and V (white), Figure S4: EDX mapping for sample **V2**@AC-ox: C (green), O (pink), Br (blue) and V (white), Figure S5: Effect of the duration of the MW irradiation on the catalytic activity of supported oxidovanadium complexes for the oxidation of cyclohexane with TBHP in MeCN, Figure S6: Total yield of KA oil obtained by the MW-assisted oxidation of cyclohexane at 80 °C, 20 W, for 2 h, supported on different carbon materials, Table S1: Summary of uses of carbon-supported oxidovanadium complexes in various catalytic reactions and major outcomes, Table S2: Supported vanadium complexes in cyclohexane oxidation and major outcomes. CCDC 2016875 and 2016876 for **1** and **2** contain the supplementary crystallographic data for this paper. These data can be obtained free of charge from The Cambridge Crystallographic Data Center via www.ccdc.cam.ac.uk/data_request/cif.
