# Supplementary material for: Oxido- and Dioxido-Vanadium(V) Complexes Supported on Carbon Materials: Reusable Catalysts for the Oxidation of Cyclohexane
Source: Nanomaterials (Basel). 2021 May 31;11(6):1456. doi: 10.3390/nano11061456 (PMC8230237; doi:10.3390/nano11061456)
Supplement: Supplementary file 1 [file nanomaterials-11-01456-s001.zip › SI-nanomaterials-1123732_Proof.pdf]

Supplementary Information

Oxido- and dioxido-vanadium(V) complexes supported on carbon materials: reusable catalysts for the oxidation of cyclohexane

Manas Sutradhar[1,*], Marta A. Andrade[1], Sónia A. C. Carabineiro[1,2,3,*], Luísa M. D. R. S. Martins[1,*], M. Fátima C. Guedes da Silva[1] and Armando J. L. Pombeiro[1,4]

[1] Centro de Química Estrutural, Instituto Superior Técnico, Universidade de Lisboa, Av. Rovisco Pais, 1049-001 Lisboa, Portugal.
[2] Laboratory of Catalysis and Materials (LCM), Associate Laboratory LSRE-LCM, Faculty of Engineering, University of Porto, 4200-465 Porto, Portugal
[3] LAQV-REQUIMTE, Departamento de Química, Faculdade de Ciências e Tecnologia, Universidade NOVA de Lisboa, Largo da Torre, 2829-516 Caparica, Portugal.
[4] Peoples' Friendship University of Russia (RUDN University), Research Institute of Chemistry, 6 Miklukho-Maklaya Street, Moscow 117198, Russian Federation

* Correspondence: manas@tecnico.ulisboa.pt (M.S.), sonia.carabineiro@fct.unl.pt (S.A.C.C.), luisammartins@tecnico.ulisboa.pt (L.M.D.R.S.)

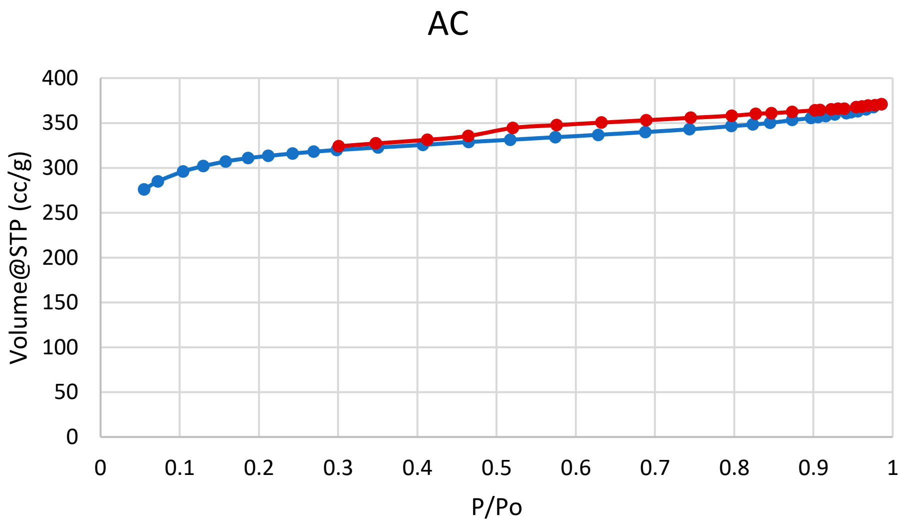

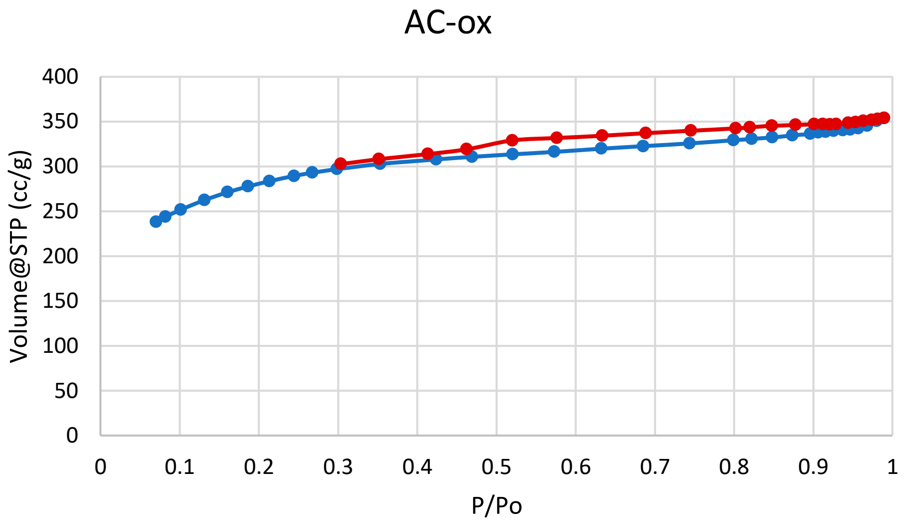

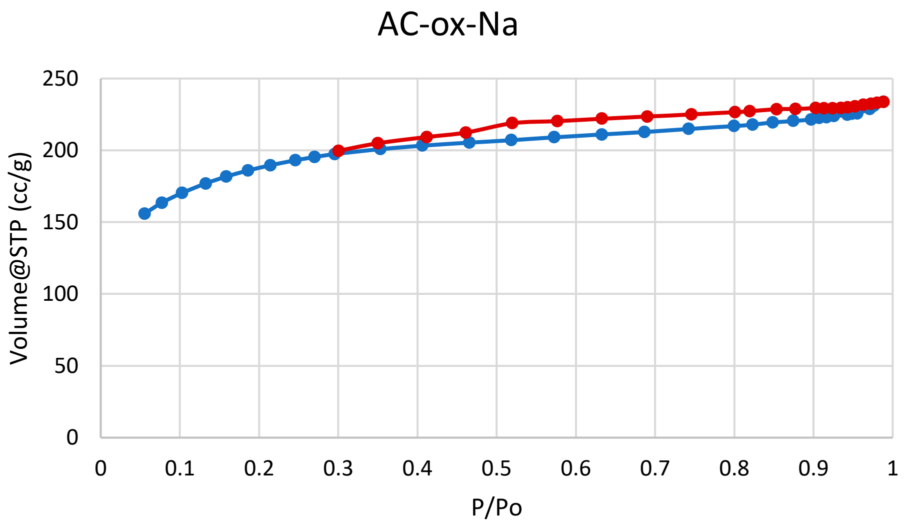

Figure S1. N_2 adsorption isotherms of AC, AC-ox, AC-ox-Na.

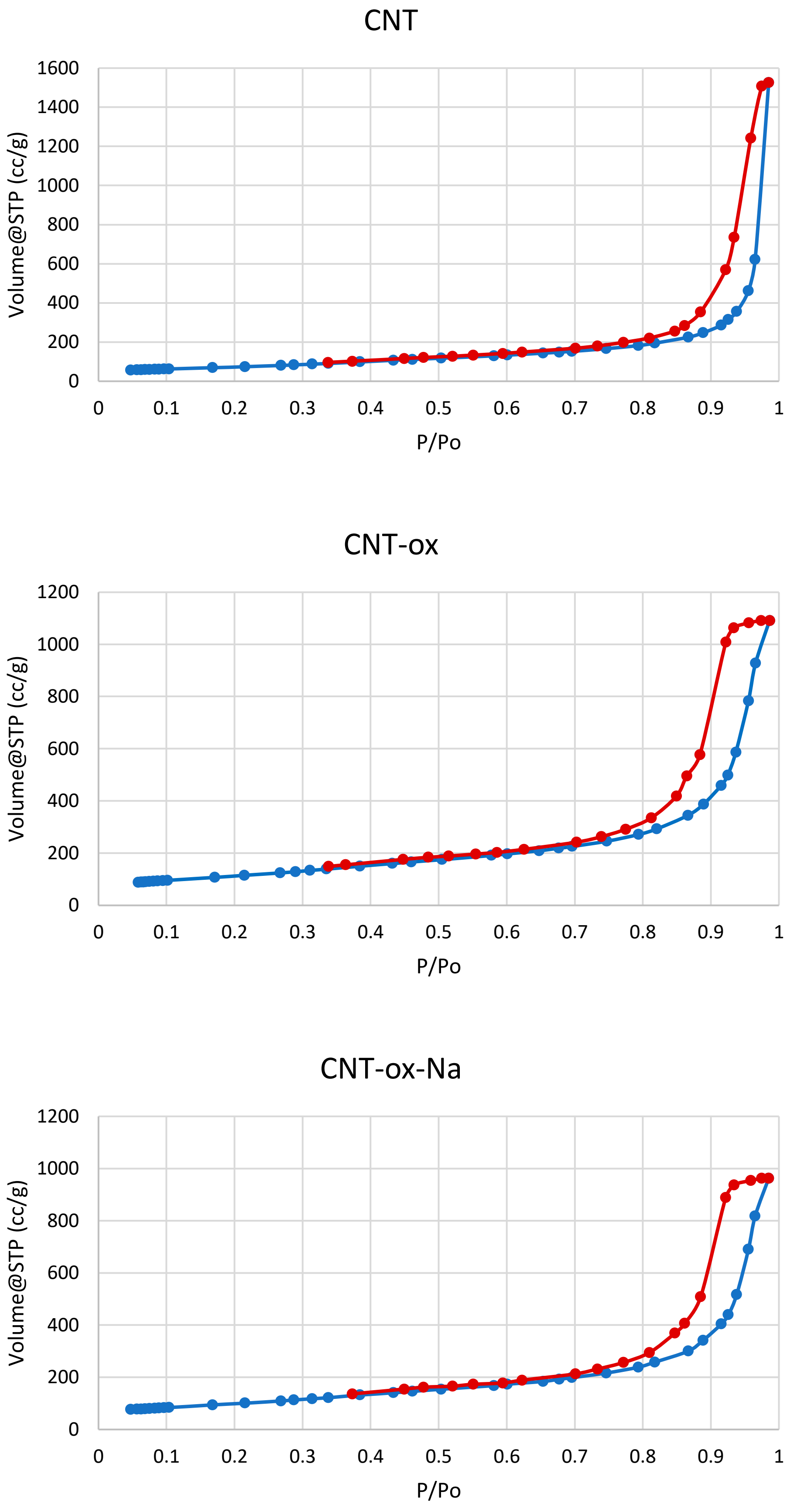

Figure S2. N_2 adsorption isotherms of CNT, CNT-ox, CNT-ox-Na.

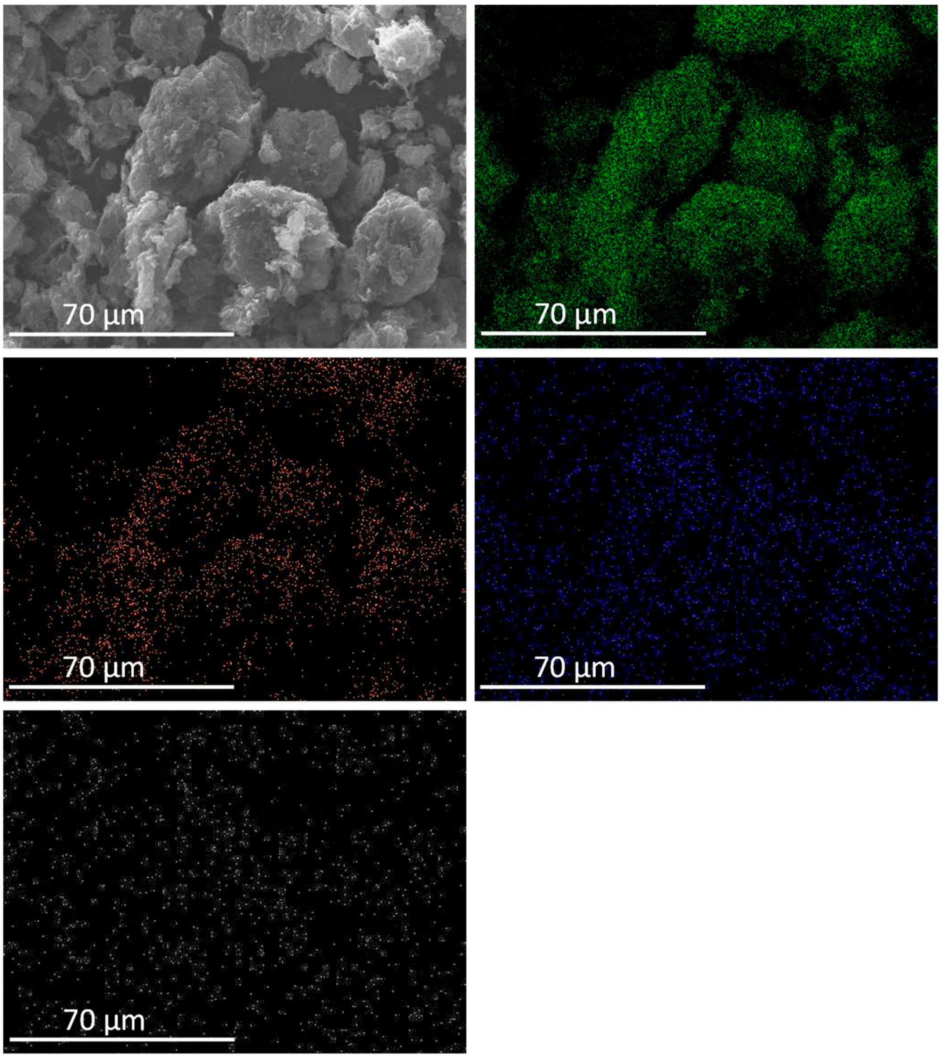

Figure S3. SEM-EDX mapping for sample **V1**@CNT-ox: C (green), O (pink), Br (blue) and V (white).

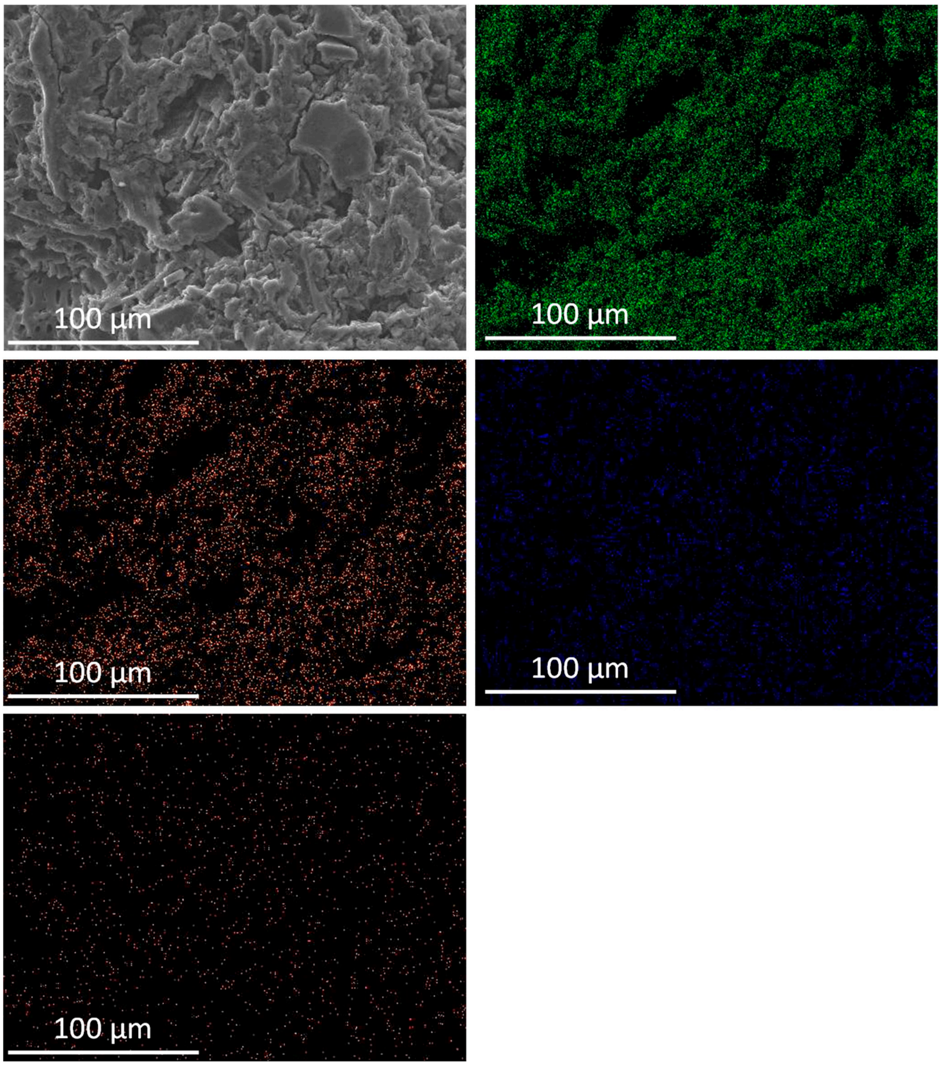

Figure S4. EDX mapping for sample **V2**@AC-ox: C (green), O (pink), Br (blue) and V (white).

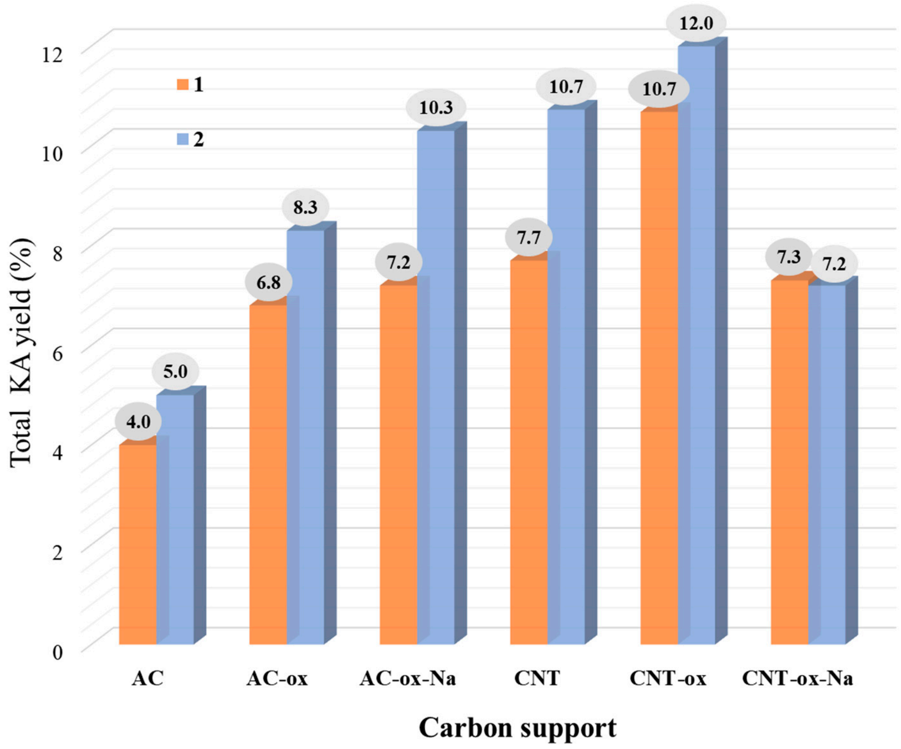

Figure S5. Total yield of KA oil obtained by the MW-assisted oxidation of cyclohexane at 80 °C, 20 W, for 2 h, supported on different carbon materials.

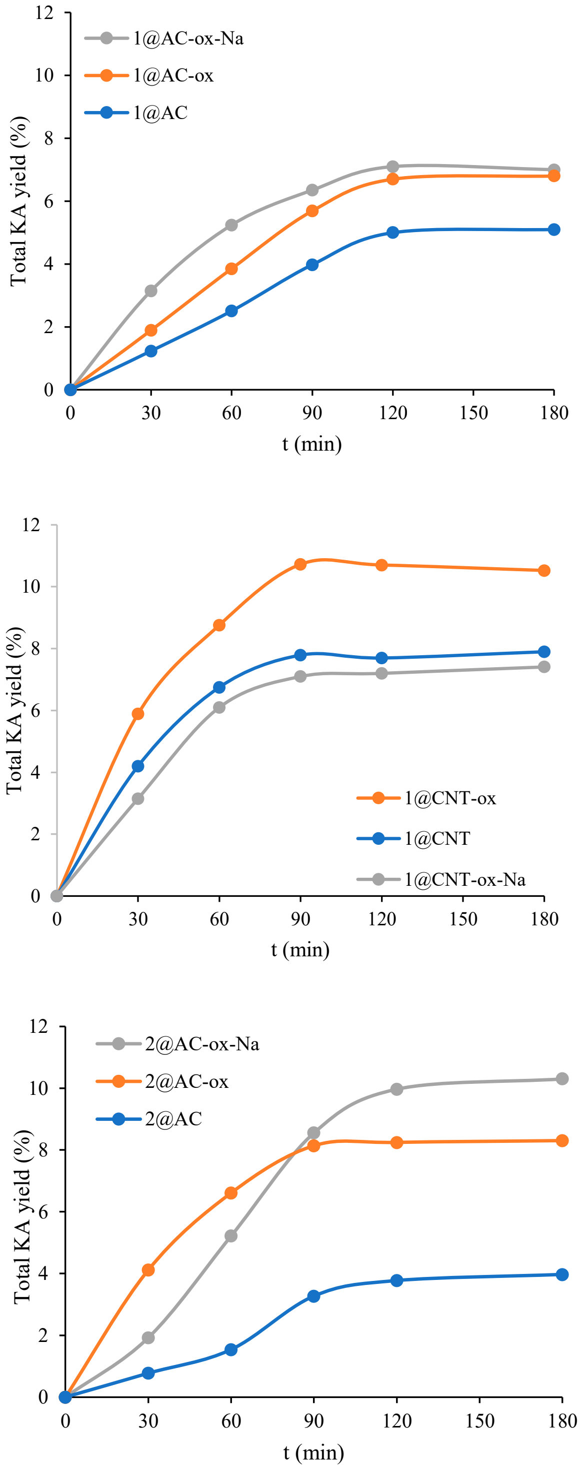

Figure S6. Effect of the duration of the MW irradiation on the catalytic activity of supported oxidovanadium complexes for the oxidation of cyclohexane with TBHP in MeCN.

Table S1. Summary of uses of carbon-supported oxidovanadium complexes in various catalytic reactions and major outcomes.

Reaction	Vanadium complexes	Carbon support	Catalytic results	Catalyst stability	Ref
1-phenylethanol oxidation	Oxovanadium (V)	Functionalized MWCNT, AC and CX	96 % acetophenone yield, > 98% selectivity	90 % of the catalytic activity was observed by the 10th cycle.	[1]
Oxidation of alcohols	Oxovanadium Schiff base	Graphene nanosheets	96 % of benzohydrazone	Efficient recycling of the catalyst over six runs.	[2]
Cyclooctene oxidation	Hydroxyl functionalized oxovanadium(IV) Schiff-base	Modified MWCNT	Cyclooctene epoxide (08.6%), cyclooctene-1-ol (52.5%) and cyclooctene-1-one (28.9%).	Reused for three times without neither loss of activity or selectivity.	[3]
Styrene oxidation	Oxovanadium(IV) acetylacetonate	Amino-modified CMK-3	66.7 % yield of styrene oxide with a selectivity of 70.5 %	A decrease of 10 % in conversion and < 10 % selectivity to styrene oxide were reported.	[4]
Styrene epoxidation	Oxovanadium(IV) salen	Amino-modified graphene oxide	18.5 % styrene epoxide, and 21% selectivity	Three recycling runs for the catalyst with a 10 % conversion drop and slight selectivity decrease (< 5%).	[5]
	Oxovanadium(IV) salen	Carbon-coated Fe_3O_4 hybridized with graphene	54.6 % yield of styrene epoxide with 59.2% selectivity	For two cycles, almost no leaching was observed; both yields and selectivity were maintained.	[6]
	Oxovanadium(II) Schiff base	Amino-functionalized graphene oxide	16.7 % yield of styrene epoxide with 20.7 % selectivity	No significant loss of activity and selectivity after successive runs (<5 %).	[7]
Epoxidation of oleic acid	Oxovanadium Schiff base	Functionalized graphene oxide	98.9 yield of the epoxide with 99.1 % selectivity	The catalysts could be successfully recycled and reused for six runs without a significant decrease in activity or selectivity.	[8]
Epoxidation of geraniol	Oxovanadium(IV) acetylacetonate	CMK-3	Conversions > 98% and 98-99% selectivity for 2,3-epoxygeraniol	For two cycles, almost no leaching was observed; both yields and selectivity were maintained.	[9]
Oxidation of p-chlorobenzenethiol	Oxovanadium (IV) complex	Amino functional microporous organic nanotube frameworks	100 % conversion was reported with 98 % selectivity.	The material was recycled more than 8 times with no apparent decrease in catalytic activity	[10]

Table S2. Supported vanadium complexes in cyclohexane oxidation and major outcomes.

Vanadium complexes	Support	Yield (%)			Selectivity to KA oil (%)	Catalyst stability	Ref
		A	K	O			
Oxidovanadium(V)	Functionalized AC and CNT	7.0	5.0	-	~100	Activity drop of 28 % by the 4th cycle.	This work
Functionalised oxidovanadium(IV) Schiff-base	Modified MWCNTs	62.5	8.9	7.5	90.5	No leaching was observed.	[11]
Oxidovanadium(IV)	PDMS	4.7	1.0	-	~100	The activity of the recycled catalyst was much lower.	[12]
V-scorpionate complex	MOR zeolite	7.9	43.9	-	~100	Loss of activity in the recycling tests: 18 % in the 4th cycle.	[13]
Vanadium phosphate	Mesoporous KIT-6 silica	4.9	8.6	5.8	69.9	A conversion decrease of 30 % was observed by the 5th cycle.	[14]
$VOSO_4$	HTS (TiO_2-modified material)	0.1	2.7	13.4	21.7	-	[15]
Vanadium phosphorus oxide (VPO)	SBA-15 and TUD-1 silicas	1.0	30.0	-	~100	-	[16]

A- cyclohexanol; K- cyclohexanone; O- others; AC-activated carbon; CNT- carbon nanotubes.

References

1. Sutradhar, M.; Martins, L.M.D.R.S.; Carabineiro, S.A.C.; Guedes da Silva, M.F.C.; Buijnsters, J.G.; Figueiredo, J.L.; Pombeiro, A.J.L. Oxidovanadium(V) Complexes Anchored on Carbon Materials as Catalysts for the Oxidation of 1-Phenylethanol. *ChemCatChem* **2016**, *8*, 2254–2266, doi:10.1002/cctc.201600316.

2. Mungse, H.P.; Verma, S.; Kumar, N.; Sain, B.; Khatri, O.P. Grafting of oxo-vanadium Schiff base on graphene nanosheets and its catalytic activity for the oxidation of alcohols. *J. Mater. Chem.* **2012**, *22*, 5427–5433, doi:10.1039/c2jm15644j.

3. Salavati-Niasari, M.; Badiei, A.; Saberyan, K. Oxovanadium(IV) salophen complex covalently anchored to multi-wall carbon nanotubes (MWNTs) as heterogeneous catalyst for oxidation of cyclooctene. *Chem. Eng. J.* **2011**, *173*, 651–658, doi:10.1016/j.cej.2011.07.023.

4. Wang, X.; Wu, S.; Li, Z.; Yang, X.; Hu, J.; Huo, Q.; Guan, J.; Kan, Q. Oxovanadium(IV), copper(II) or cobalt(II) acetylacetone complexes immobilized on amino-functionalized CMK-3 for the aerobic epoxidation of styrene. *Appl. Organomet. Chem.* **2015**, *29*, 698–706, doi:10.1002/aoc.3353.

5. Su, H.; Wu, S.; Li, Z.; Huo, Q.; Guan, J.; Kan, Q. Co(II), Fe(III) or VO(II) Schiff base metal complexes immobilized on graphene oxide for styrene epoxidation. *Appl. Organomet. Chem.* **2015**, *29*, 462–467, doi:10.1002/aoc.3316.

6. Li, Z.; Wu, S.; Zheng, D.; Ding, H.; Wang, X.; Yang, X.; Huo, Q.; Guan, J.; Kan, Q. Enhanced aerobic epoxidation of styrene with copper(II), cobalt(II), iron(III), or

oxovanadium(IV) salen complexes immobilized onto carbon-coated Fe3O4 nanoparticles hybridized with graphene sheets. *Chempluschem* **2014**, *79*, 716–724, doi:10.1002/cplu.201300424.

7. Li, Z.; Wu, S.; Ding, H.; Lu, H.; Liu, J.; Huo, Q.; Guan, J.; Kan, Q. Oxovanadium(iv) and iron(iii) salen complexes immobilized on amino-functionalized graphene oxide for the aerobic epoxidation of styrene. *New J. Chem.* **2013**, *37*, 4220–4229, doi:10.1039/c3nj00982c.

8. Verma, S.; Aila, M.; Kaul, S.; Jain, S.L. Immobilized oxo-vanadium Schiff base on graphene oxide as an efficient and recyclable catalyst for the epoxidation of fatty acids and esters. *RSC Adv.* **2014**, *4*, 30598–30604, doi:10.1039/c4ra03454f.

9. Dorbes, S.; Pereira, C.; Andrade, M.; Barros, D.; Pereira, A.M.; Rebelo, S.L.H.; Araújo, J.P.; Pires, J.; Carvalho, A.P.; Freire, C. Oxidovanadium(IV) acetylacetonate immobilized onto CMK-3 for heterogeneous epoxidation of geraniol. *Microporous Mesoporous Mater.* **2012**, *160*, 67–74, doi:10.1016/j.micromeso.2012.03.041.

10. Zhang, H.; Zhou, M.; Xiong, L.; He, Z.; Wang, T.; Xu, Y.; Huang, K. Oxo-vanadium (IV) complex supported by microporous organic nanotube frameworks: A high selective heterogeneous catalyst for the oxidation of thiols to disulfides. *Microporous Mesoporous Mater.* **2018**, *255*, 103–109, doi:10.1016/j.micromeso.2017.07.041.

11. Salavati-Niasari, M.; Bazarganipour, M. Synthesis, characterization and liquid phase oxidation of cyclohexane with hydrogen peroxide over oxovanadium(IV) Schiff-base tetradendate complex covalently anchored to multi-wall carbon nanotubes (MWNTs). *Bull. Korean Chem. Soc.* **2009**, *30*, 355–362, doi:10.5012/bkcs.2009.30.2.355.

12. Silva, T.F.S.; Leod, T.C.O.M.; Martins, L.M.D.R.S.; Guedes Da Silva, M.F.C.; Schiavon, M.A.; Pombeiro, A.J.L. Pyrazole or tris(pyrazolyl)ethanol oxo-vanadium(IV) complexes as homogeneous or supported catalysts for oxidation of cyclohexane under mild conditions. *J. Mol. Catal. A Chem.* **2013**, *367*, 52–60, doi:10.1016/j.molcata.2012.10.024.

13. Ottaviani, D.; Van-Dúnem, V.; Carvalho, A.P.; Martins, A.; Martins, L.M.D.R.S. Eco-friendly cyclohexane oxidation by a V-scorpionate complex immobilized at hierarchical MOR zeolite. *Catal. Today* **2019**, 1–8, doi:10.1016/j.cattod.2019.09.034.

14. Rezaei, M.; Najafi Chermahini, A.; Dabbagh, H.A. Green and selective oxidation of cyclohexane over vanadium pyrophosphate supported on mesoporous KIT-6. *Chem. Eng. J.* **2017**, *314*, 515–525, doi:10.1016/j.cej.2016.12.009.

15. Zhong, W.; Qiao, T.; Dai, J.; Mao, L.; Xu, Q.; Zou, G.; Liu, X.; Yin, D.; Zhao, F. Visible-light-responsive sulfated vanadium-doped TS-1 with hollow structure: Enhanced photocatalytic activity in selective oxidation of cyclohexane. *J. Catal.* **2015**, *330*, 208–221, doi:10.1016/j.jcat.2015.06.013.

16. Santra, C.; Shah, S.; Mondal, A.; Pandey, J.K.; Panda, A.B.; Maity, S.; Chowdhury, B. Synthesis, characterization of VPO catalyst dispersed on mesoporous silica surface and catalytic activity for cyclohexane oxidation reaction. *Microporous Mesoporous Mater.* **2016**, *223*, 121–128, doi:10.1016/j.micromeso.2015.10.046.
